# Why Will Polymers Win the Race for Solid‐State Batteries?

**DOI:** 10.1002/advs.202510481

**Published:** 2025-08-07

**Authors:** Zhiyong Li, Sisi Peng, Lu Wei, Xin Guo

**Affiliations:** ^1^ School of Materials Science and Engineering State Key Laboratory of Material Processing and Die & Mould Technology Huazhong University of Science and Technology Wuhan 430074 P. R. China; ^2^ Solid Ionic Power Technology (Wuhan) Co., Ltd. Wuhan 430000 P. R. China

**Keywords:** oxide, polymer, solid electrolyte, solid‐state battery, sulfide

## Abstract

Solid‐state batteries (SSBs) promise to revolutionize energy storage by offering enhanced safety, higher energy density, and improved cycle lifespan over conventional lithium‐ion batteries. Among the various solid electrolytes, polymers stand out for their unique combination of processability, mechanical compliance, and chemical versatility. This review explores why polymers are poised to lead the race toward commercial SSBs. Their intrinsic advantages—such as superior interfacial contact with electrodes, tunable ionic conductivity, and compatibility with scalable manufacturing methods—as well as the key technical challenges they face, including limited thermal stability, narrow electrochemical windows, and interfacial degradation, are examined. This study highlights emerging solutions from recent research, including polymer molecular design, polymer–ceramic composites, and in situ polymerization strategies. In contrast to oxide and sulfide systems, which face significant barriers in cost, manufacturability, and integration, polymer‐based electrolytes offer a realistic and economically viable path to large‐scale deployment. With continuing advances in materials design and industrial processing, polymers are not only competitive—they are leading the transition to next‐generation solid‐state batteries.

## Graphical abstract

Polymer‐based electrolytes are leading solid‐state battery contenders by balancing processability, mechanical compliance, and interfacial compatibility. Their tunable chemistry enables scalable, cost‐effective manufacturing, overcoming oxide/sulfide limitations. While ionic conductivity and electrochemical stability challenges remain, advances in molecular design, polymer–ceramic composites, and in situ polymerization offer solutions. Polymers are the most practical and economically viable route to high‐performance solid‐state batteries.

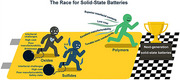

## 1 Introduction

The development of solid‐state batteries (SSBs) has emerged as a critical technological frontier in the quest for safer, higher‐energy‐density, and longer‐lasting energy storage systems.^[^ ^1^, ^2^, ^3^ ^]^ By replacing the flammable liquid electrolytes used in conventional lithium‐ion batteries (LIBs) with solid‐state ionic conductors, SSBs offer the potential for enhanced safety, expanded electrochemical stability windows, and the integration of lithium metal anodes—all of which contribute to higher theoretical performance limits.^[^ ^4^, ^5^, ^6^ ^]^

Solid electrolytes fall broadly into three material classes: oxides, sulfides, and polymers. Each class presents a distinct combination of properties, advantages, and disadvantages, summarized in **Table** **1** and elaborated in subsequent sections. For instance, oxide electrolytes exhibit high chemical stability but suffer from brittleness and poor interfacial contact with electrodes.^[^ ^10^ ^]^ Sulfide electrolytes offer excellent ionic conductivity and better interfacial contact, yet they are highly sensitive to moisture and require stringent processing conditions.^[^ ^11^ ^]^ In contrast, polymer electrolytes have traditionally been dismissed due to their low room‐temperature conductivity but excel in terms of processability, flexibility, and interfacial compatibility.^[^ ^12^ ^]^

**Table 1:** Key properties of solid electrolytes.^[^ ^7^, ^8^, ^9^ ^]^

Property	Oxides	Sulfides	Polymers
Ionic conductivity	Moderate (10^−4^–10^−3^ S cm^−1^)	High (10^−3^–10^−2^ S cm^−1^)	Low to moderate (10^−6^–10^−3^ S cm^−1^)
Electrochemical stability	Excellent	Moderate	Tunable
Mechanical properties	Brittle	Better than oxides	Flexible and soft
Interface compatibility	Poor with electrodes	Better than oxides	Excellent
Processability	Requires high‐temperature sintering	Requires inert atmosphere	Easy (solution/melt processable)
Moisture sensitivity	Low	Very high	Low
Manufacturing cost	High	Very high	Low

However, as SSB technologies progress from laboratory‐scale prototypes toward commercial viability, the metrics for success are shifting. Beyond electrochemical performance, commercial success will hinge on pragmatic factors such as high manufacturing throughput, interfacial robustness, supply chain maturity, and cost‐efficiency across the full production cycle.^[^ ^13^ ^]^ From this perspective, many of the inherent limitations of inorganic electrolytes become increasingly problematic when scaled, as discussed in Section 2.

This review takes an opinionated stance: polymer electrolytes—often perceived as the underdogs in the race toward solid‐state commercialization—are, in fact, the most promising platform for scalable, manufacturable SSBs. Through a detailed examination of technical challenges, production constraints, and materials integration in Sections 3 and 4, we argue that polymers offer the best balance of performance and practicality for next‐generation solid‐state energy storage.

## 2 Inorganic Electrolytes

### 2.1 Advantages and Disadvantages

Inorganic or ceramic electrolytes, such as oxides and sulfides, have garnered significant attention in the SSB research due to their high ionic conductivities and, in the case of oxides, wide electrochemical stability windows.^[^ ^14^ ^]^ These materials have enabled laboratory demonstrations of SSBs, including cells with lithium metal anodes and high‐voltage cathodes. For example, polyacrylonitrile–Li_7_La_3_Zr_2_O_12_ (LLZO) nanofiber hybrids have achieved an ionic conductivity of ≈0.3 mS cm^−1^ at 25 °C,^[^ ^15^ ^]^ chloride‐based electrolytes have been synthesized at processing temperatures below 400 °C,^[^ ^16^ ^]^ and Ta‐doped LLZO with in situ‐formed LiF‐rich interphases has enabled Li||Li symmetric cells to operate for over 600 cycles at 1 mA cm^−2^.^[^ ^17^ ^]^ However, the performance metrics achieved under idealized lab‐scale conditions often obscure fundamental limitations that pose serious challenges for large‐scale manufacturing and commercial deployment.

Oxide‐based solid electrolytes, such as garnet‐type LLZO, are prized for their chemical stability and compatibility with high‐voltage cathode materials. Their ability to resist decomposition in contact with both electrodes makes them attractive from a durability standpoint.^[^ ^18^ ^]^ However, oxides are inherently brittle, making them difficult to process into defect‐free, thin, and flexible layers. Moreover, they exhibit poor interfacial contact with both cathodes and lithium metal anodes due to their rigidity and low surface conformability. Realizing functional interfaces often requires the use of external pressure, soft buffer layers, or high‐temperature treatments—none of which are easily scalable.^[^ ^19^ ^]^ In addition, high‐temperature sintering (often above 1000 °C) is required to achieve the desired phase purity and densification,^[^ ^20^ ^]^ adding significant cost and complexity to the fabrication process.

Sulfide‐based electrolytes, such as Li_10_GeP_2_S_12_ (LGPS), are recognized for their exceptionally high ionic conductivities,^[^ ^11^ ^]^ in some cases exceeding those of liquid electrolytes. Their mechanical softness allows for interfacial contact better than oxides, facilitating ion transport across electrode/electrolyte interfaces.^[^ ^21^ ^]^ However, sulfides come with severe moisture sensitivity, reacting readily with atmospheric water to produce toxic hydrogen sulfide (H_2_S) gas.^[^ ^22^ ^]^ This necessitates the use of fully inert environments throughout material synthesis, cell assembly, and even storage—substantially increasing operational and safety costs. Furthermore, sulfide electrolytes are often electrochemically unstable against lithium metal, and require protective coatings or interlayers to prevent decomposition.^[^ ^23^ ^]^

While both oxide and sulfide electrolytes offer good ionic transport properties, they are accompanied by critical drawbacks in mechanical robustness, chemical stability, and process compatibility. These issues, as further detailed in Section 2.2, collectively hinder the scalability and manufacturability of inorganic SSBs and challenge their viability for mass‐market applications.

### 2.2 Technical Challenges

#### 2.2.1 Solid/Solid Contact Issues

Among the most fundamental challenges in the development of SSBs is the issue of solid/solid interfacial contact. Unlike conventional lithium‐ion batteries, which rely on liquid electrolytes that naturally infiltrate porous electrodes to form continuous and conformal interfaces, SSBs must achieve effective ionic and electronic contact through rigid, often brittle, solid materials. The lack of intimate and stable contact across electrode/electrolyte interfaces directly compromises electrochemical performance, mechanical integrity, and long‐term cycling stability.

**Figure 1:**
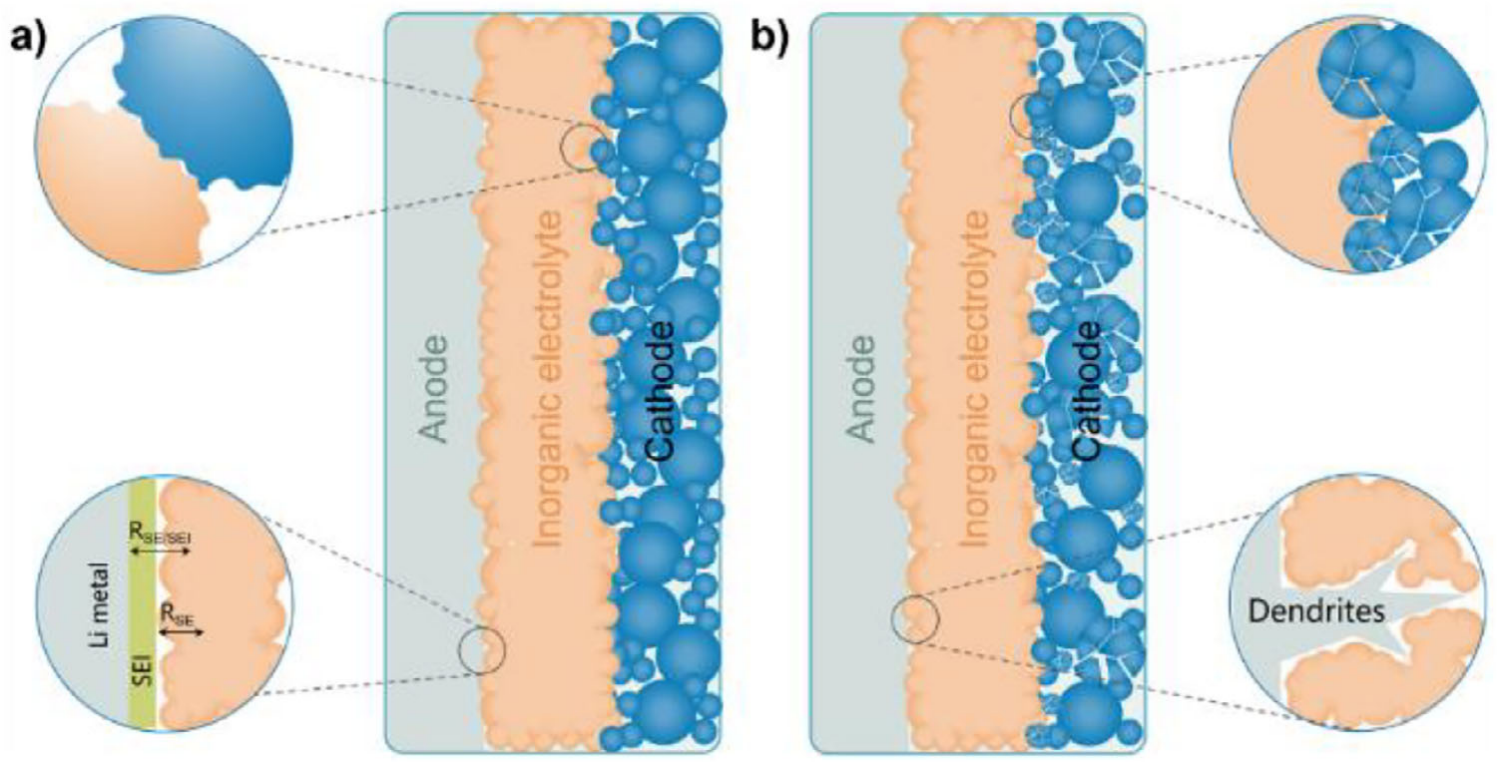
Battery failure arising from mechanical‐thermal property mismatch between inorganic electrolytes and electrode materials. a) Initial operation stage showing interface integrity. b) Advanced degradation stage demonstrating contact loss and crack propagation.

At the heart of the problem is the inherent mismatch in mechanical and thermal properties between the solid electrolytes and the active electrode materials.^[^ ^24^ ^]^ During fabrication and subsequent battery cycling, materials expand and contract due to temperature fluctuations and lithium insertion/extraction (**Figure** **1**a). These strains are poorly accommodated in solid–solid systems, often resulting in delamination, interfacial void formation, or microcracking. Such defects lead to increased interfacial resistance, nonuniform current distribution, and in severe cases, localized lithium plating or dendrite formation (Figure 1b).

Oxide‐based SSBs, which typically use rigid ceramic electrolytes like LLZO, are particularly prone to contact degradation. These materials do not deform plastically and therefore cannot maintain conformal contact with electrodes without the application of external pressure or the use of compliant interlayers.^[^ ^25^ ^]^ However, applying constant stack pressure adds complexity to cell packaging and is impractical for large‐format commercial cells.

Sulfide‐based SSBs, by contrast, exhibit better deformability and can form improved initial contact with electrodes. Nevertheless, they still face challenges related to mechanical degradation during cycling, especially in systems where high‐capacity materials such as lithium metal or silicon are employed.^[^ ^26^ ^]^ The mismatch in expansion behavior can still induce contact loss or the formation of interfacial degradation layers, which impede the ion transport and reduce the cycle lifespan.

Furthermore, the interfaces themselves may undergo chemical reactions, particularly in sulfide systems, forming passivating interphases that further increase resistance and limit ion transport.^[^ ^27^ ^]^ Engineering stable, low‐resistance interfaces thus require not only mechanical optimization but also chemical compatibility, often involving coatings, buffer layers, or dopant strategies that add complexity to materials design and processing.

Solid/solid contact issues are a multidimensional challenge, affecting almost every performance metric of SSBs—from ionic conductivity and rate capability to mechanical durability and lifespan. Without effective strategies to ensure durable and scalable interfacial contact, the full potential of solid‐state battery technology cannot be realized.

#### 2.2.2 Equipment Overhaul Requirements

The manufacturing of SSBs, particularly those incorporating ceramic‐based electrolytes, requires a fundamental rethinking of equipment design and process architecture. Unlike conventional LIB production—which relies on slurry coating, solvent evaporation, and liquid electrolyte filling—SSB fabrication demands entirely new equipment configurations, often involving high‐pressure consolidation, controlled‐atmosphere processing, and novel stacking or lamination technologies.

For oxide‐based SSBs, which utilize rigid and brittle ceramic electrolytes such as LLZO, production workflows must incorporate high‐temperature sintering furnaces (typically >1000 °C) to achieve densification and phase purity. Such equipment is rarely found in existing battery manufacturing lines, and is more commonly associated with ceramic or semiconductor processing. In addition, the integration of oxide ceramics into functional battery stacks often requires hot pressing or spark plasma sintering to ensure intimate contact between the electrolyte and electrode layers—techniques that are not compatible with current roll‐to‐roll manufacturing paradigms.^[^ ^28^ ^]^ These processes also involve batch‐type operations, which are inherently lower in throughput and more expensive to scale.

Sulfide‐based SSBs, while more mechanically compliant and processable at lower temperatures, come with their own unique set of equipment requirements. Because sulfide materials are highly sensitive to moisture and oxygen, all stages of their synthesis, electrode integration, and cell assembly must be performed in dry rooms or inert‐atmosphere gloveboxes. This includes powder mixing, calendaring, layer stacking, and even cell sealing.^[^ ^29^ ^]^ Such facilities are significantly more expensive to construct and maintain than those used in conventional LIB production, and few commercial‐scale environments exist today that are fully compatible with sulfide chemistries.

Moreover, both oxide and sulfide SSBs require advanced stacking and packaging technologies to accommodate rigid or fragile components and to maintain interfacial pressure throughout cycling. This may involve the development of flexible current collectors, compliant interlayers, or precision lamination systems that can preserve alignment and structural integrity under mechanical and thermal stress. Existing LIB equipment—optimized for pouch, cylindrical, or prismatic formats with liquid infiltration—is ill‐suited for such tasks.

In practical terms, transitioning from the LIB production to the SSB manufacturing involves substantial capital investment in new tooling, environmental control systems, and automation schemes. The equipment overhaul is not incremental but rather systemic, touching nearly every aspect of the value chain from materials handling to cell finalization. As such, equipment requirements represent a major barrier to the industrialization of solid‐state battery technologies, especially when considered alongside the immaturity of supporting supply chains and the high cost of raw materials.

#### 2.2.3 Process Incompatibility with Existing Production Lines

A key obstacle to the commercialization of oxide‐ and sulfide‐based SSBs is their incompatibility with the existing LIB manufacturing infrastructure. Today's multi‐gigawatt‐scale battery factories are highly optimized for liquid electrolyte cells, relying on well‐established processes such as slurry casting, roll‐to‐roll manufacturing, electrolyte filling, and vacuum sealing. In contrast, SSBs—particularly those using inorganic solid electrolytes—require entirely different process flows, materials handling protocols, and equipment configurations.

For oxide‐based SSBs, the integration of brittle ceramic layers such as LLZO necessitates precision tape casting, high‐temperature sintering, and pressure‐assisted lamination, none of which are standard in LIB production lines.^[^ ^30^ ^]^ These operations often involve low‐throughput batch processes rather than continuous roll‐to‐roll manufacturing, which severely limits production scalability and drives up costs. Moreover, achieving the necessary interfacial contact between rigid oxides and electrode materials demands novel assembly techniques, such as hot pressing or spark plasma sintering, that are incompatible with current LIB plant layouts and automation systems.

Sulfide‐based SSBs, while more deformable than oxides, come with their own processing challenges. These materials are highly sensitive to moisture and release toxic gases upon contact with air, requiring fully inert processing environments throughout fabrication. This includes mixing, calendaring, and stacking under dry or inert conditions—a significant departure from the relatively mild environmental requirements of the LIB manufacturing. In addition, sulfide electrolytes do not adhere well to electrodes without the use of soft interlayers or high‐pressure consolidation, further complicating electrode–electrolyte integration steps.^[^ ^31^ ^]^

Retrofitting existing gigafactories to accommodate these solid‐state processes is not straightforward. The changes needed are not incremental but foundational, requiring a complete overhaul of materials flow, equipment layout, safety infrastructure, and quality control protocols. In effect, commercial‐scale SSB production would demand the construction of entirely new manufacturing lines—or even purpose‐built facilities—with specialized equipment and environmental controls.

This process‐level discontinuity is a fundamental barrier to the near‐term commercialization of solid‐state battery technologies based on oxide and sulfide electrolytes. Unless scalable and process‐compatible architectures or validated hybrid approaches emerge, the transition from laboratory prototypes to industrial‐scale production will remain slow and capital‐intensive.

#### 2.2.4 Supply Chain Disruption

SSBs based on oxides and sulfides face significant supply chain disruptions, stemming from both upstream material constraints and downstream processing requirements. From precursor chemicals to specialized synthesis and fabrication equipment, the infrastructure needed to support commercial‐scale production remains underdeveloped. This immaturity poses a critical bottleneck to the timely and cost‐effective deployment of the SSB technology.

For oxide‐based SSBs, key electrolyte materials such as LLZO require rare or strategically sensitive elements (e.g., lanthanum, zirconium), whose extraction and purification are controlled by a few global suppliers.^[^ ^32^ ^]^ Moreover, oxide electrolytes demand high‐temperature sintering (>1000 °C) and sophisticated ceramic processing techniques, necessitating equipment and expertise not widely available outside niche sectors. These manufacturing steps not only increase capital expenditure but also constrain scalability due to low process yields.

Sulfide‐based SSBs introduce a different set of vulnerabilities. Their electrolytes—such as Li_10_GeP_2_S_12_ and Li_6_PS_5_Cl—depend on materials like high‐purity phosphorus and germanium, with the latter being scarce and geopolitically sensitive.^[^ ^33^ ^]^ Sulfide powders are also chemically reactive and release toxic H_2_S gas upon exposure to moisture, requiring controlled‐atmosphere processing facilities that are expensive to operate. The lack of standardized, industrial‐grade synthesis protocols for these compounds further complicates procurement and production planning.

In both cases, battery‐grade lithium must be supplied in specific chemical forms (e.g., lithium sulfide or lithium nitrate), which differ from those used in conventional lithium‐ion battery production.^[^ ^34^ ^]^ This divergence limits the compatibility with existing lithium supply chains and increases dependency on customized refining and synthesis routes.

A complete reconfiguration of the supply chain—from mining to materials processing to cell fabrication—would be required to support the large‐scale commercialization of oxide‐ and sulfide‐based SSBs. However, building such a vertically integrated and diversified supply infrastructure is a long‐term endeavor and cannot be realized within the timeframe typically expected for commercial technology rollouts. Until these systemic gaps are addressed, supply chain limitations will remain a formidable barrier to the widespread adoption of solid‐state battery technologies based on oxides and sulfides.

#### 2.2.5 Raw Material Costs

The raw material costs associated with oxide‐ and sulfide‐based SSBs remain a significant economic hurdle. High‐purity LLZO and sulfide‐based electrolytes such as lithium thiophosphates require specialized precursors and stringent synthesis conditions that drive up production costs, particularly when scaled to industrial volumes.

For oxide‐based systems, the reliance on elements such as lanthanum and zirconium introduces inherent cost volatility. Both are relatively rare and subject to geopolitical supply risks, with lanthanum largely derived from rare‐earth mining operations and zirconium production concentrated in a few regions. Furthermore, LLZO and other garnet‐type electrolytes must be synthesized with strict compositional control and sintered at high temperatures, often necessitating dopants like tantalum or gallium to stabilize the crystal structure—further inflating material costs.^[^ ^35^ ^]^

**Figure 2:**
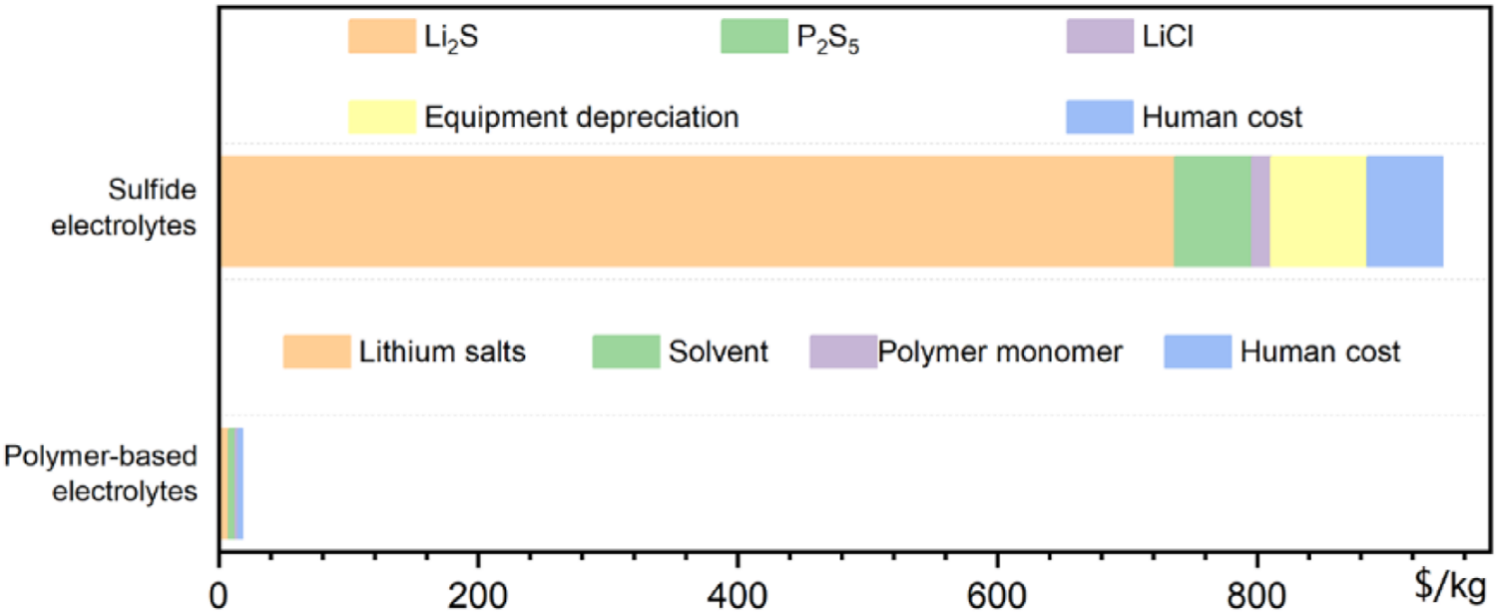
Production cost of sulfide electrolytes and polymer‐based electrolytes.^[^ ^33^, ^39^ ^]^ Li_2_S is priced at over $700 kg^−1^, posing significant cost challenges for the industrialization of sulfide electrolytes.Reproduced with permission.^[^ ^33^ ^]^ Coptright 2025, Elsevier. Reproduced with permission.^[^ ^39^ ^]^ Copyright 2025, Springer Nature.

Sulfide‐based electrolytes, such as Li_6_PS_5_Cl or Li_10_GeP_2_S_12_, are no less expensive (**Figure** **2**). Their synthesis depends on high‐purity phosphorus and sulfur compounds, and in some formulations, germanium, a critical element with limited availability and a high cost per kilogram.^[^ ^36^ ^]^ Germanium is particularly problematic due to its co‐dependence on zinc mining and the fact that a significant portion of global refining capacity resides in politically sensitive regions.^[^ ^37^ ^]^ Even sulfur, despite being abundant, requires purification to battery‐grade levels, which adds processing steps and associated costs.^[^ ^38^ ^]^

Adding to the challenge is the requirement for battery‐grade lithium in specialized forms, such as lithium chloride or lithium sulfide, which are not typically produced at scale in the current lithium supply ecosystem. These variants require additional chemical transformation steps, further stretching the cost structure compared to conventional lithium‐ion battery chemistries. In contrast, polymers exhibit significantly lower costs (Figure 2),^[^ ^39^ ^]^ rendering them particularly attractive for large‐scale applications, as further discussed in Section 3.

Overall, the high cost of raw materials for oxides and sulfides not only impedes their price competitiveness with incumbent lithium‐ion technologies but also presents a challenge for future cost reduction. Without breakthroughs in alternative, lower‐cost electrolyte materials or significant improvements in synthesis efficiency and precursor utilization, raw material expenses will continue to be a critical limiting factor in the economic viability of solid‐state battery technologies.

#### 2.2.6 Safety Concerns

While SSBs are often promoted for their enhanced safety relative to conventional lithium‐ion systems, the reality is more nuanced—particularly when considering sulfide‐based chemistries. These systems present a unique set of chemical and operational hazards that complicate their handling, storage, and manufacturing.

**Figure 3:**
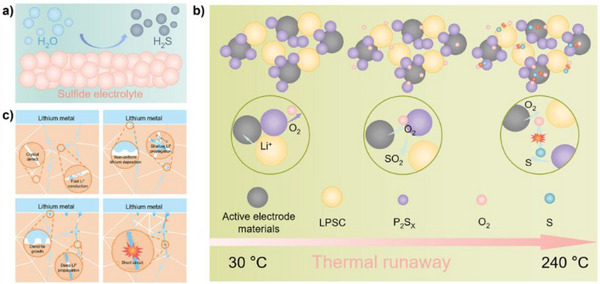
Safety challenges in inorganic electrolytes. a) Sulfide electrolytes react with trace moisture to generate toxic H_2_S gas.^[^ ^22^ ^]^ Reproduced with permission.^[^ ^22^ ^]^ Copyright 2025, John Wiley and Sons.b) Sulfide electrolytes undergo side reactions with the active electrode materials during Li^+^ transport, generating O_2_. Subsequent exothermic reactions between O_2_ and P_2_S_x_ raise the battery temperature. Above 240 °C, this positive feedback loop accelerates, increasing thermal runaway risk.^[^ ^40^ ^]^ Reproduced with permission.^[^ ^40^ ^]^ Copyright 2023, Royal Society of Chemistry. c) In oxide electrolytes, Li dendrites propagate through defects and grain boundaries.^[^ ^41^ ^]^ Crystal defects facilitate rapid ion transport, while the electrolyte's electronic conductivity promotes Li^+^ reduction, forming metallic Li nuclei within the bulk. Simultaneous electron transfer at the electrolyte/electrode interface promotes inhomogeneous Li deposition. Eventually, these internal Li nuclei bridge with Li dendrites, creating short‐circuit pathways that compromise the battery safety.Reproduced with permission.^[^ ^41^ ^]^ Copyright 2024, Elsevier.

The primary safety concern associated with sulfide solid electrolytes lies in their reactivity with moisture. Compounds such as Li_6_PS_5_Cl and Li_10_GeP_2_S_12_ are highly sensitive to atmospheric humidity.^[^ ^22^ ^]^ Upon exposure to ambient conditions, they can undergo hydrolysis, releasing H_2_S gas—a toxic and flammable compound (**Figure** **3**a). Even trace levels of moisture can trigger measurable off‐gassing, posing significant risks to worker safety and necessitating strict inert‐atmosphere protocols during synthesis, cell assembly, and even storage of raw materials. The installation and maintenance of such protective environments (e.g., dry rooms or gloveboxes filled with inert gases like argon or nitrogen) introduce substantial capital and operational costs, in addition to complicating process scalability.

Furthermore, some sulfide compositions exhibit low thermal stability and can undergo exothermic decomposition or catching fire at elevated temperatures, which raises concerns about their behavior under abuse conditions (e.g., thermal runaway or short circuits) (Figure 3b).^[^ ^40^ ^]^ Although SSBs eliminate flammable organic liquid electrolytes, sulfide‐based systems can still sustain combustion and release noxious gases when compromised, which presents significant safety concerns.

In contrast, oxide‐based solid electrolytes are generally more chemically stable and nonflammable. However, they are not entirely free of safety considerations. Their high brittleness and sensitivity to mechanical stresses can result in microcracking and loss of interfacial contact, which may cause localized overpotentials, dendrite formation, or thermal hotspots during cycling.^[^ ^41^ ^]^ In extreme cases, such defects may precipitate internal short circuits or structural failure (Figure 3c).

Thus, while solid‐state architectures offer a pathway to safer batteries in principle, the material choices involved—particularly for sulfides—introduce new categories of risk that must be carefully managed through materials engineering, packaging strategies, and rigorous operational controls.

Collectively, these obstacles—ranging from solid/solid interfacial challenges and equipment incompatibilities to raw material costs, safety hazards, and underdeveloped supply chains—are increasingly recognized as potential showstoppers for the mass deployment of oxide‐ and sulfide‐based SSBs. The challenges are not isolated issues that can be resolved through piecemeal engineering; rather, they are deeply interconnected and systemic, requiring coordinated advances across materials science, process engineering, supply logistics, and manufacturing infrastructure. In this context, incremental improvements are unlikely to suffice. Without disruptive innovation or a fundamental shift in the material or architectural paradigm, these hurdles will continue to delay the widespread commercialization of inorganic solid‐state battery technologies.

## 3 Polymer‐Based Electrolytes

Polymer‐based electrolytes represent a distinct and increasingly promising class of solid‐state ionic conductors, which are generally classified into three categories: (1) solid polymer electrolytes (SPEs), consisting solely of polymers and lithium salts; (2) composite polymer electrolytes (CPEs), formed by incorporating inorganic fillers into SPEs; and (3) quasi‐solid/gel polymer electrolytes (GPEs), which contain immobilized liquid phases. Materials such as polyethylene oxide (PEO), polycarbonate‐based polymers, and more recently, block copolymers and composite systems, offer several advantages over their oxide and sulfide counterparts—particularly in the areas of interfacial compatibility, mechanical compliance, and processability.

One of the most compelling attributes of polymer‐based electrolytes is their excellent interfacial contact with both electrodes. Unlike ceramics, polymers are soft and conformable, allowing them to form intimate interfaces without the need for high stack pressures or complex interfacial engineering. This flexibility is especially beneficial when used with lithium metal anodes or composite cathodes, where volumetric changes during cycling can otherwise lead to contact loss or interfacial delamination in more rigid systems.^[^ ^42^ ^]^

While room‐temperature ionic conductivities of conventional polymer electrolytes—such as PEO/LiTFSI—are typically lower (10^−6^–10^−4^ S cm^−1^) than those of sulfides or oxides,^[^ ^43^ ^]^ significant progress has been made in recent years. Composite polymer electrolytes, which incorporate ceramic or ionically conductive fillers, and block copolymer architectures have been shown to improve both conductivity and mechanical strength.^[^ ^44^ ^]^ Some hybrid systems now approach or exceed the 10^−3^ S cm^−1^ threshold, making them viable for room temperature operation.^[^ ^45^ ^]^

A key differentiator of polymer‐based electrolytes is their compatibility with the existing LIB manufacturing infrastructure. They can be processed using low‐temperature, scalable techniques such as solution casting, extrusion, and roll‐to‐roll processing.^[^ ^46^ ^]^ These methods are well‐established in the battery industry, enabling cost‐effective integration without the need for inert atmospheres, high‐temperature sintering, or specialized equipment. This compatibility dramatically lowers the barrier to scale‐up, especially when compared to the infrastructure overhaul required for oxide‐ and sulfide‐based systems.

**Figure 4:**
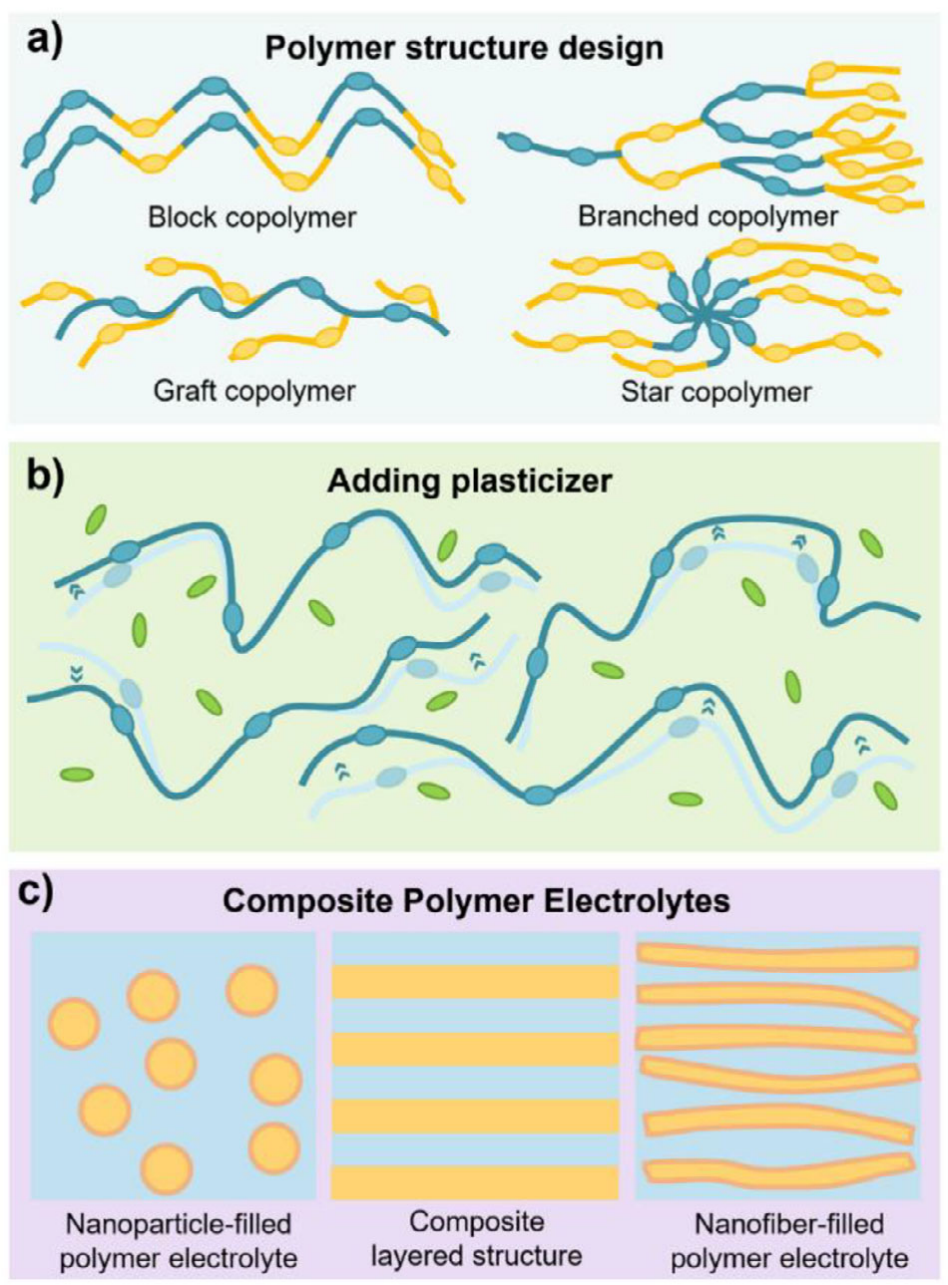
Strategies for modulating the properties of polymer‐based electrolytes (ionic conductivity, electrochemical window, thermal stability, etc.). a) Molecular architecture control: Precise design of polymer topologies (block, graft, branched, or star copolymers) enables tailored electrolyte properties through strategic manipulation of chain structure and chemical composition. b) Functional plasticizer engineering: Specially designed plasticizers not only increase polymer chain mobility to boost ionic conductivity but also simultaneously expand the electrochemical stability window. c) Hybrid composite: Strategically formulated polymer‐inorganic composites leverage synergistic effects, where careful optimization of both the polymer matrix and inorganic filler morphology enables superior electrolyte performances.

Polymers are also chemically tunable, allowing their properties to be tailored through backbone modifications, cross‐linking, or blending with functional additives (**Figure** **4**).^[^ ^47^ ^]^ This opens up a versatile design space where conductivity, mechanical strength, thermal behavior, and electrochemical stability can be optimized in parallel. Importantly, most polymer systems exhibit low moisture sensitivity and low toxicity, simplifying materials handling and reducing safety‐related operating costs.^[^ ^48^ ^]^

Although polymer electrolytes have historically been limited by ionic conductivity constraints, recent innovations in materials design and composite strategies are closing the performance gap. When balanced against their superior manufacturability, mechanical adaptability, and compatibility with lithium metal, polymer electrolytes emerge as the most practical and scalable path toward solid‐state battery commercialization.

### 3.1 Process Compatibility and Scalability

Among the various classes of solid electrolytes, polymer systems stand out for their compatibility with scalable manufacturing processes. Unlike oxide and sulfide electrolytes, which often require significant retooling of production infrastructure and strict environmental controls, polymers offer a low‐barrier entry point for SSB manufacturing, especially when leveraging existing LIB production lines.

**Figure 5:**
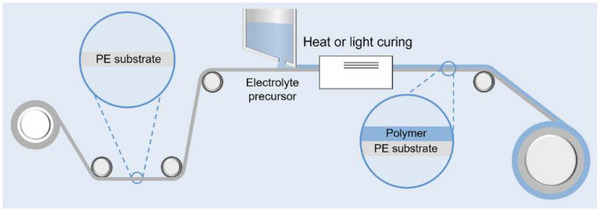
In the production of polymer electrolyte membranes, an electrolyte precursor solution is cast onto a polyethylene (PE) substrate and subsequently cured to form a continuous electrolyte membrane. Such a process is compatible with the existing roll‐to‐roll battery manufacturing processes.

Polymers are inherently well‐suited to membrane processing (**Figure** **5**), with fabrication methods such as tap casting, and roll‐to‐roll lamination already established in the battery industry. These techniques operate at relatively low temperatures (<150 °C) and are compatible with ambient or dry‐room environments, significantly reducing capital expenditures and operational complexity.^[^ ^49^ ^]^ This is in stark contrast to oxide‐based systems, which often require high‐temperature sintering (>1000 °C), and sulfide‐based systems, which demand stringent moisture‐ and oxygen‐free handling to prevent degradation and toxic byproduct formation.

Moreover, polymers enable monolithic, continuous processing across the full battery stack—solid electrolyte, electrode interfaces, and encapsulation layers—facilitating integrated, high‐throughput manufacturing. This streamlining reduces step count, material loss, and alignment errors, all of which are challenges in the batch‐processed, pressure‐intensive approaches required for inorganic electrolytes. Polymers also offer greater geometric adaptability, accommodating irregular form factors, flexible substrates, or micro‐batteries for wearables and embedded systems.

From a supply chain perspective, the polymer ecosystem benefits from mature industrial infrastructure.^[^ ^50^ ^]^ Many polymer hosts and additives used in electrolyte formulations are commercially available at scale and do not rely on niche or geopolitically sensitive elements such as lanthanum, zirconium, or germanium. This supply stability, coupled with the ability to synthesize polymers in bulk using well‐established chemical routes, supports cost‐effective scale‐up and mitigates risk during commercialization.

**Figure 6:**
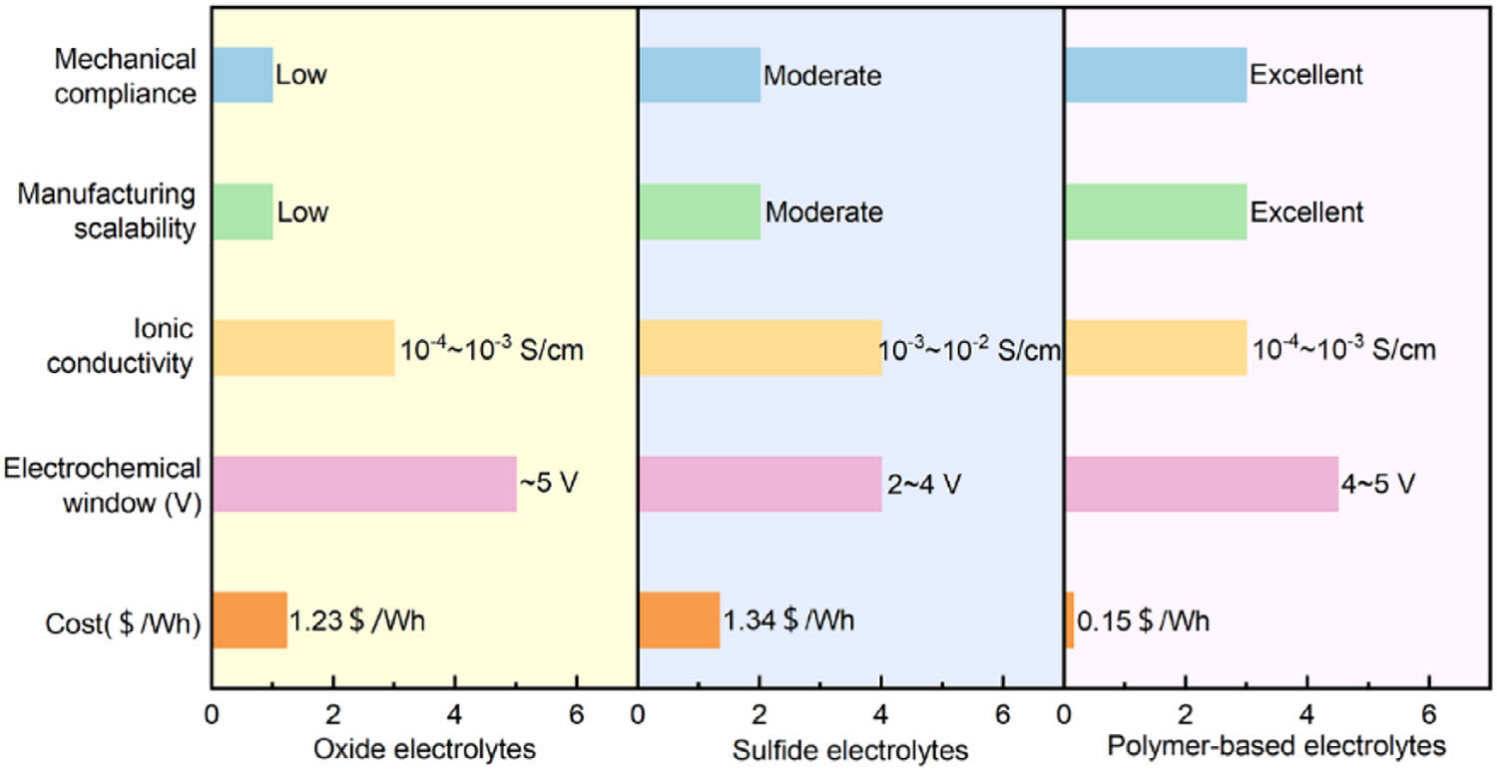
Figure‐of‐merit plot—conductivity at 25 °C versus electrochemical window and cost—for polymers, oxides, and sulfides.^[^ ^51^, ^52^ ^]^

While performance trade‐offs remain—particularly in terms of ionic conductivity and thermal stability—the process advantages of polymers are both immediate and decisive. The compatibility of polymers with existing manufacturing infrastructure substantially reduces the need for capital reinvestment (**Figure** **6**),^[^ ^51^, ^52^ ^]^ which also translates into high manufacturing yields. As a result, polymer‐based SSBs hold a practical advantage in the transition from laboratory‐scale development to gigafactory‐level production. As the industry moves toward mass‐market adoption, these manufacturing attributes may prove more determinative than marginal gains in conductivity or energy density alone.

### 3.2 Major Challenges and Solutions

#### 3.2.1 Low Ionic Conductivity

Low ionic conductivity has long been recognized as a primary limitation of polymer‐based solid electrolytes, especially at ambient temperatures. Traditional solid polymer electrolytes, such as PEO complexes with lithium salts, exhibit conductivities in the range of 10^−7^–10^−6^ S cm^−1^ at room temperature—several orders of magnitude below the levels required for high‐performance battery applications.^[^ ^53^ ^]^ This bottleneck has historically constrained the practical deployment of polymer electrolytes in SSBs.

Recent advances in polymer chemistry and materials design have begun to shift this landscape (Figure 4). One strategy involves modifying the polymer backbone to disrupt crystallinity and enhance segmental mobility, thereby significantly improving ionic conductivity.^[^ ^54^ ^]^ Another strategy employs polymer molecular engineering, such as copolymerization and blending, which enable decoupling of mechanical strength and ionic transport.^[^ ^55^, ^56^, ^57^ ^]^ In these systems, one block provides a mechanically robust matrix while the other hosts fast‐ion‐conducting domains. This architectural control allows for optimized microphase separation, resulting in continuous ion‐conducting pathways and improved conductivity.

The incorporation of plasticizers, such as plastic crystal,^[^ ^58^ ^]^ eutectic mixtures,^[^ ^59^ ^]^ ionic liquids,^[^ ^60^ ^]^ or low‐molecular‐weight solvents,^[^ ^61^ ^]^ has also proven effective in enhancing segmental mobility and thus ion transport. Liquid plasticizers alter the ion transport mechanism compared to all‐solid‐state systems, while compromising mechanical and thermal stability. Multiscale structural design has been explored to overcome the trade‐off between ionic conductivity and mechanical strength. For example, poly(ether‐urethane)‐based polymer electrolytes with dynamic covalent disulfide bonds and hydrogen bonds were designed,^[^ ^62^ ^]^ the intermolecular interaction can not only effectively anchor solvent molecules, but also ensure that the electrolyte maintains excellent mechanical properties. Meanwhile, inorganic–polymer composites have also emerged as another promising solution by combining the flexibility and processability of polymers with the high ionic conductivity and electrochemical stability of ceramic fillers. Fillers rich in Lewis acid sites can confine solvent molecules, while the formation of percolation networks enhances Li^+^ transport and simultaneously toughens the electrolyte.^[^ ^63^, ^64^, ^65^, ^66^ ^]^ In situ polymerization enables the uniform distribution of plasticizers and strong interfacial mechanical cohesion by offering better control over the polymer structure, thereby facilitating ionic conduction while preserving the mechanical integrity of the polymer framework.^[^ ^67^ ^]^

While room‐temperature ionic conductivities of pure polymers remain below those of liquid electrolytes, the gap is narrowing. Many polymer‐based systems now demonstrate conductivities exceeding 10^−4^ S cm^−1^ at room temperature (**Table** **2**). Thus, although low ionic conductivity remains a critical challenge, it is increasingly being addressed through intelligent material design and targeted application‐specific performance tuning.

**Table 2:** Strategies for improving ionic conductivity of polymers.

Strategy	Electrolyte system	Ionic conductivity [mS cm^−1^]	Refs.
End‐group functionalization	Cellulose phthalate	1.09	[54]
Copolymerization	Fluorinated polyesters	0.59	[55]
Copolymerization	PVEC/DAC/MBA	0.66	[56]
Polymer blending	PVDF‐HFP/PTFEP	0.3	[57]
Introducing plasticizers	PBA/PEGDA/SN	4.1	[58]
Introducing plasticizers	PEGDA/UPyMA/NML	0.34	[59]
Introducing plasticizers	12‐HOA/IL	1.09	[60]
Introducing plasticizers	POM/FDMA	2.5	[61]
Composite electrolytes	PVDF/MMT	1.08	[63]
Composite electrolytes	PVDF/BTO	0.82	[64]
Composite electrolytes	P(VDF‐TrFE)/Li_6_PS_5_Cl	1.2	[65]
Composite electrolytes	PEG‐HDMI/Zr‐MOF	0.57	[66]

#### 3.2.2 Insufficient Electrochemical Window

The electrochemical stability window of polymer electrolytes is another critical challenge that constrains their applicability in high‐voltage SSB systems. Most conventional polymer electrolytes, including PEO‐based systems, are inherently unstable above ≈3.8–4.0 V versus Li/Li^+^.^[^ ^68^ ^]^ This limitation impedes their compatibility with high‐energy cathode materials such as nickel‐rich layered oxides (e.g., NCM811) or high‐voltage spinels, which operate at voltages approaching or exceeding 4.3 V. At these voltages, oxidative decomposition of the polymer backbone or salt anion can lead to parasitic reactions, electrolyte degradation, and the formation of resistive interphases, ultimately compromising battery performance and lifespan.

To address this, several strategies have been developed. One approach involves the rational design of novel polymer backbones that are intrinsically more resistant to oxidative degradation. For instance, increasing the length of the extended alkyl chains of polymers,^[^ ^69^ ^]^ and reducing Li^+^–polymer interactions and strengthen anion‐polymer interactions to form anion‐rich solvation structures.^[^ ^70^ ^]^ Alternatively, fluorinated polymers have shown enhanced oxidative stability due to the presence of electron‐withdrawing groups that lower the highest occupied molecular orbital (HOMO) energy levels;^[^ ^71^ ^]^ the ion‐bridged strategy utilizes Zn^2+^─O coordination to stabilize lone pairs of ethereal oxygen, which suppresses polyether electrolyte oxidation as well.^[^ ^72^ ^]^

Another strategy is to use the residual monomer after polymerization to derive stable cathode electrolyte interphase (CEI) layers to further inhibit electrolyte oxidation. These interlayers act as kinetic barriers, suppressing direct chemical contact and reducing interfacial reactivity. Such class of monomers, exhibiting high HOMO values, have demonstrated efficacy in stabilizing polymer‐cathode interfaces at high operating voltages.^[^ ^73^, ^74^ ^]^

In addition, hybrid electrolyte architecture—where a more stable ceramic or polymer‐inorganic composite layer interfaces with the cathode—are gaining traction. These designs enable a cascade of stability, where each layer is chemically tuned to match the electrochemical potential of its adjacent component, effectively widening the operational voltage window of the full cell.^[^ ^75^, ^76^, ^77^ ^]^

While achieving stable operation above 4.2 V remains a non‐trivial task, these engineering approaches are rapidly improving the voltage tolerance of polymer electrolytes (**Table** **3**). Continued progress in this area will be essential to unlocking the full energy potential of SSBs employing high‐voltage cathodes.

**Table 3:** Strategies for improving electrochemical stability window (ESW) of polymers.

Strategy	Electrolyte system	ESW (V vs Li/Li^+^)	Cathode	Refs.
Extend alkyl chain	PDOX	≈4.7	4.5 V NCM811	[69]
Dipole competitive effect	PDMA	≈5.5	4.5 V NCM523	[70]
Cross‐linking	PETPTA/TAEP	≈4.9	4.6 V LNCMO	[71]
Ion‐bridging strategy	Zn‐IBPE	≈5.0	4.5 V LCO	[72]
Constructing stable CEIs	PETEA/DAP	≈5.8	5.0 V LRO	[73]
Constructing stable CEIs	HFBA/MBAM	≈4.8	4.5 V NCM622	[74]
Composite electrolytes Composite electrolytes Composite electrolytes	PVDF/MoSe_2_	≈4.7	4.3 V NCM811	[75]
	PDOL/LLZTO	≈4.7	4.5 V NCM811	[76]
	PEO/TIO	≈4.75	4.3 V NCM811	[77]

#### 3.2.3 Limited Thermal Stability and Safety

Thermal stability is a critical safety parameter in the design of SSBs, particularly for applications such as electric vehicles and grid storage, where exposure to elevated temperatures can be unavoidable. Many conventional polymer electrolytes begin to degrade thermally at temperatures above ≈100 °C, leading to softening, chain scission, or even exothermic decomposition.^[^ ^78^ ^]^ This degradation not only compromises mechanical integrity and ionic conductivity but also increases the risk of thermal runaway in the event of a short circuit or thermal abuse.

The root cause lies in the organic nature of polymer electrolytes, whose backbones are susceptible to thermal cleavage or oxidation. For example, PEO and other ether‐based polymers have relatively low decomposition temperatures and poor dimensional stability at elevated temperatures.^[^ ^79^ ^]^ This presents a substantial safety and reliability challenge compared to ceramic electrolytes, which are often stable beyond 300 °C.

To address these concerns, researchers are exploring several strategies to enhance the thermal robustness of polymer systems. One promising route involves thermally crosslinked polymers, in which covalent bonds form between polymer chains during or after processing to create a three‐dimensional network.^[^ ^80^ ^]^ These crosslinked structures exhibit significantly improved thermal stability, mechanical strength, and shape retention under heat stress, without sacrificing ion transport properties when designed appropriately.

Another major development is the incorporation of inorganic fillers or the formation of polymer–ceramic composites. By embedding thermally stable ceramic particles—such as Al_2_O_3_, SiO_2_, or garnet‐type oxides—into the polymer matrix, these composites benefit from improved heat resistance, suppressed polymer chain mobility, and enhanced flame retardancy.^[^ ^81^ ^]^ Some composite systems also exhibit synergistic improvements in ionic conductivity and interfacial stability.

In addition, the development of fluorinated and phosphorus‐containing polymers introduces flame retardancy and thermal stability, helping mitigate safety concerns that often accompany high ionic conductivity formulations.^[^ ^82^ ^]^ The flame‐retardant performance shows concentration dependence: 2 wt% fluoropolymer addition reduces self‐extinguishing time from 54 to 3.4 s g^−1^ (94% decrease), while 5 wt% loading achieves non‐flammability.^[^ ^83^ ^]^ This improvement is attributed to strong solvent–polymer interactions that significantly lower the liquid's volatility, and fluorination that suppresses the formation of hydrogen free radicals. These materials offer a route toward safer electrolytes without relying entirely on structural reinforcements.

Material optimization of polymers also enhances the safety. For example, the electrolyte synthesized from polymerizable lithium perfluoropinacolatoaluminate exhibits superior flame‐retardant properties.^[^ ^84^ ^]^ By leveraging fluorine's synergistic flame‐retardant mechanism to quench free‐radical chain reactions, the system raised the thermal runaway onset temperature (*T*_2_) of a 2.8 Ah pouch cell to 185 °C—a 63.7% increase over conventional liquid electrolytes (113 °C). To further mitigate thermal runaway triggered by high‐temperature electrode crosstalk, the polymer matrix is engineered with reactive unsaturated double bonds that spontaneously form a dense crosslinked network upon thermal activation.^[^ ^85^ ^]^ This network effectively retards the diffusion kinetics of Li‐ions and flammable gases, raising the *T*_2_ of a 1.1 Ah pouch cell to 250 °C—significantly higher than the 148 °C observed with liquid electrolytes.

Furthermore, the combination of an ethylene carbonate‐free electrolyte with in situ polymerization enabled multi‐level protection in Ah‐scale quasi‐solid‐state batteries: (1) the polymer framework suppressed exothermic reactions between LiPF_6_ and the lithiated anode; (2) a LiF/POF_3_ layer formed via fluorinated monomer decomposition delayed oxygen release at the high‐nickel cathode; and (3) the 3D polymer network inhibited electrode crosstalk at elevated temperatures. Consequently, the 2.2 Ah pouch cell achieved a *T*_2_ of 248 °C.^[^ ^86^ ^]^ These findings demonstrate that multi‐scale design of polymer‐based electrolytes substantially enhances the battery safety.

**Figure 7:**
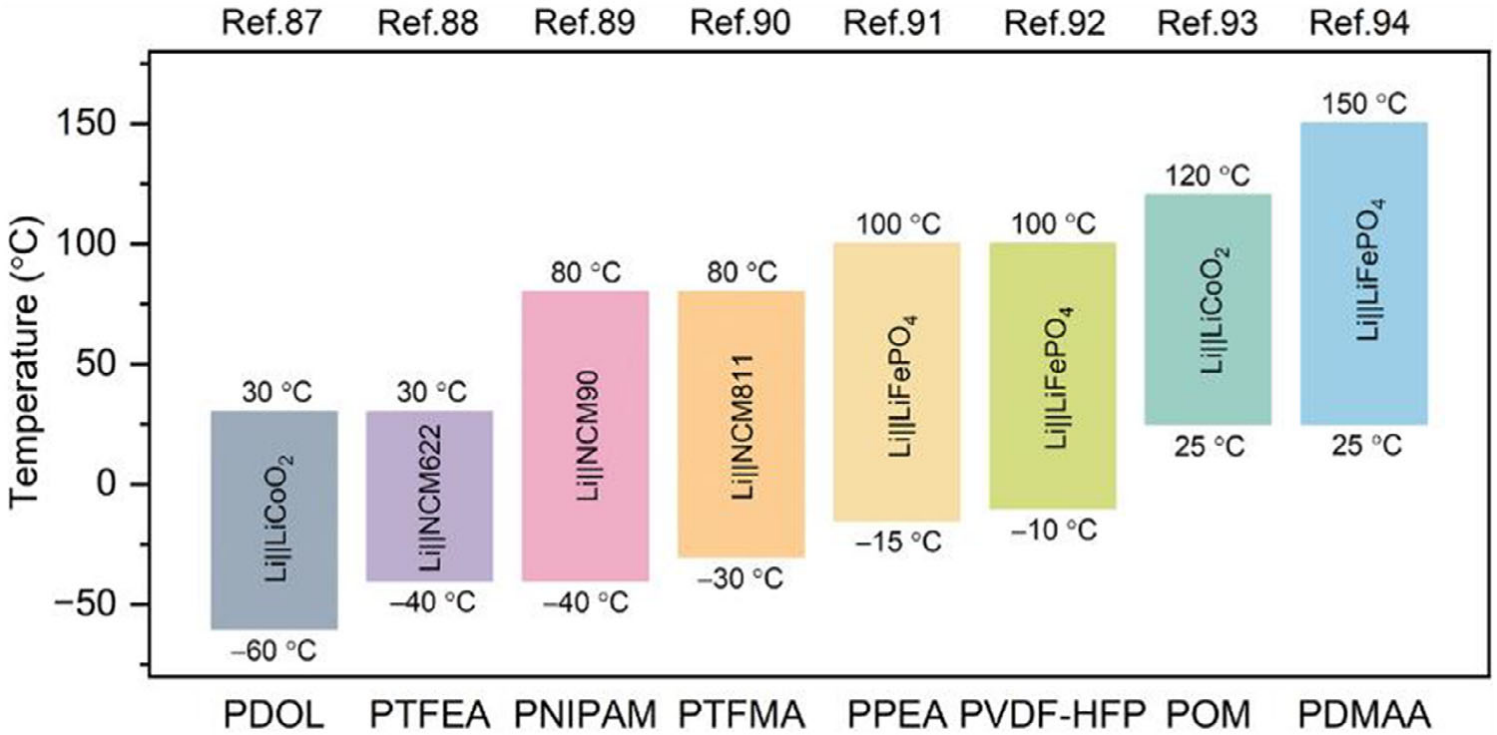
The state‐of‐the‐art wide‐temperature performance of polymer‐based solid‐state batteries.

These advancements are extending the thermal operating limits of polymer electrolytes, enabling safer battery operation over a wide temperature range from −60 to 150 °C, as illustrated in **Figure** **7**.^[^ ^87^, ^88^, ^89^, ^90^, ^91^, ^92^, ^93^, ^94^ ^]^ As the performance requirements for SSBs continue to rise, especially in harsh operating environments, thermally resilient polymer architectures will be key to ensuring both safety and reliability.

#### 3.2.4 Interfacial Stability

Achieving stable and low‐resistance interfaces remains one of the most persistent challenges in the development of SSBs. Unlike liquid electrolytes, which readily wet electrode surfaces and conform to microscopic features, solid electrolytes—especially ceramics—struggle with poor physical contact, leading to high interfacial impedance, mechanical delamination, and unstable electrochemical performance over time. This issue is especially pronounced at the interface between the electrolyte and lithium metal anodes, where interfacial void formation and dendrite penetration are common failure modes.

**Figure 8:**
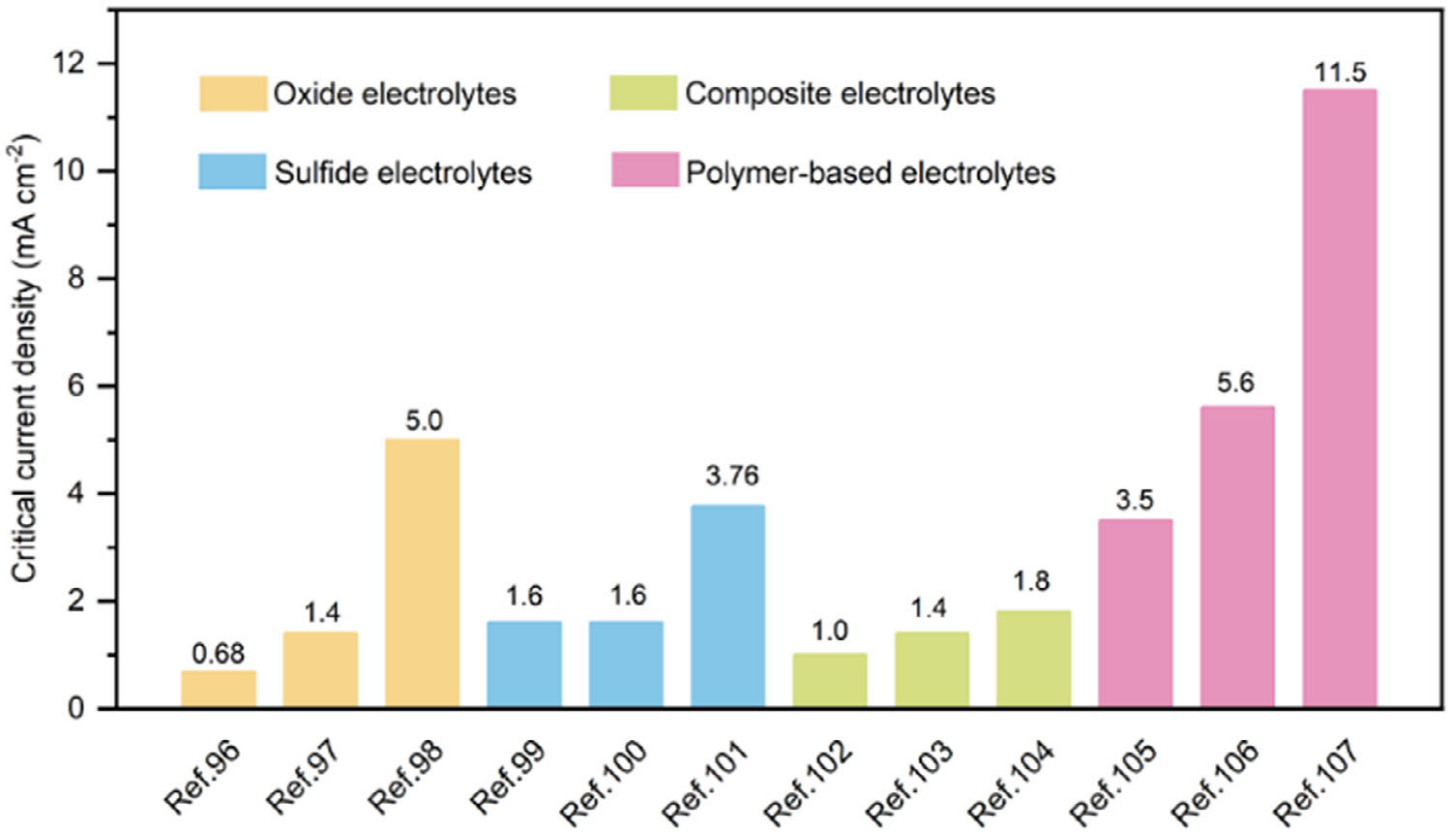
Comparison of the critical current densities.

Polymer‐based electrolytes, however, offer a key advantage in this context: their inherent ductility and viscoelasticity enable them to conform to electrode surfaces and accommodate volume changes during cycling.^[^ ^95^ ^]^ This self‐adjusting behavior helps maintain continuous interfacial contact, suppress mechanical delamination, and reduce impedance growth over time. Therefore, polymer‐based systems tend to exhibit critical current densities (**Figure** **8**, typically >3 mA cm^−2^) higher than those of composite polymer electrolytes and ceramic electrolytes.^[^ ^96^, ^97^, ^98^, ^99^, ^100^, ^101^, ^102^, ^103^, ^104^, ^105^, ^106^, ^107^ ^]^ As such, polymers are uniquely well‐suited to mitigate one of the most troublesome bottlenecks of solid‐state systems—the solid/solid interface.

**Figure 9:**
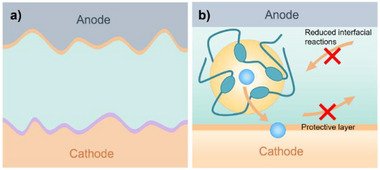
Interfacial compatibility of polymer electrolytes. a) In situ polymerization enables ultra‐conformal electrode/electrolyte interfaces with both cathode and anode, significantly reducing interfacial resistance. b) The inherent chemical stability of polymer electrolytes suppresses interfacial side reactions while simultaneously forming protective passivation layers on both electrodes.

Moreover, the ability to process polymers at low temperatures allows for in situ casting directly onto electrode surfaces, promoting excellent initial contact and reducing fabrication complexity (**Figure** **9**a).^[^ ^108^ ^]^ These features enable the formation of intimate interfaces that evolve gradually with cycling rather than catastrophically failing due to cracking or voids, as seen in more brittle solid electrolyte systems.

Nevertheless, interfacial instability can still arise from chemical incompatibility. For example, reactive lithium metal can reduce or decompose many polymers, leading to interphase formation and increased resistance. To counter this, researchers are developing interfacial engineering strategies such as thin buffer layers (e.g., lithium nitride, lithium fluoride),^[^ ^109^ ^]^ chemically stable artificial SEIs,^[^ ^110^ ^]^ and polymer surface functionalization to suppress parasitic reactions and stabilize interfaces over long‐term cycling (Figure 9b).^[^ ^111^ ^]^

While interfacial stability remains a formidable challenge for all solid‐state systems, polymers offer a uniquely adaptive and engineerable platform. Their mechanical compliance, ease of processing, and potential for interfacial tuning position them as particularly promising candidates for addressing interfacial limitations in solid‐state batteries.

While each of these technical hurdles—low ionic conductivity, narrow electrochemical stability windows, limited thermal resilience, and interfacial instability—has historically constrained the adoption of polymer‐based electrolytes, recent advances have significantly shifted the landscape. The field is benefiting from a convergence of molecular design, composite engineering, and interfacial science. While significant technical challenges remain, they are increasingly seen as tractable and are already the focus of intensive research and development efforts across both academia and industry.

## 4 Manufacturing of Polymer‐Based SSBs

### 4.1 Definition of Solid‐State Battery

The International Union of Pure and Applied Chemistry (IUPAC) defines solid polymer electrolyte as “electrically conducting solution of a salt in a polymer.”**^[^** ^112^ **^]^** This terminology explicitly classifies ionically conducting polymers as solid electrolytes despite their reliance on solution‐phase conduction mechanisms—directly challenging rigid “solid‐only” paradigms in battery discourse. In addition, as established by Tarascon and Armand,^[^ ^113^ ^]^ Manthiram et al.,^[^ ^114^ ^]^ Archer et al.,^[^ ^115^ ^]^ and others,^[^ ^116^, ^117^, ^118^, ^119^ ^]^ SSBs inherently include polymer electrolytes—whether dry or gel‐based. Therefore, the definition of SSBs refers to systems in which the electrolyte remains nonflowable under operating conditions and retains dimensional stability throughout cycling. This definition accommodates polymer electrolytes that may include plasticizers, as long as they form a nonflowable, cohesive phase—consistent with how industry and academia have described gel polymer electrolytes, quasi‐solid‐state systems.^[^ ^120^ ^]^ Accordingly, we argue that “solid‐state” should be defined not by the complete absence of liquid phase, but by the functional and mechanical properties of the electrolyte phase, particularly its ability to prevent leakage, and maintain structural integrity.

Battery innovation must prioritize tangible performance metrics—including energy density, cycling lifespan, charge/discharge rate, safety, and cost—over adherence to “solid‐only” paradigms. All‐solid‐state batteries (ASSBs) are tools, not targets, in this mission. We urge policymakers to refocus development strategies on outcome‐driven objectives through all viable electrochemical approaches.

### 4.2 Composite Electrodes

One of the defining advantages of polymer‐based SSBs is their compatibility with composite electrode architectures, where the electrolyte is intimately integrated into the electrode structure itself. Unlike ceramic electrolytes, which often require careful mechanical stacking and face challenges in achieving intimate contact with electrode particles, polymers can be easily blended, cast, or infiltrated into porous electrode scaffolds. This process compatibility enables the formation of composite electrodes, in which the electrolyte, active material, and conductive additives coexist in a percolating network.

**Figure 10:**
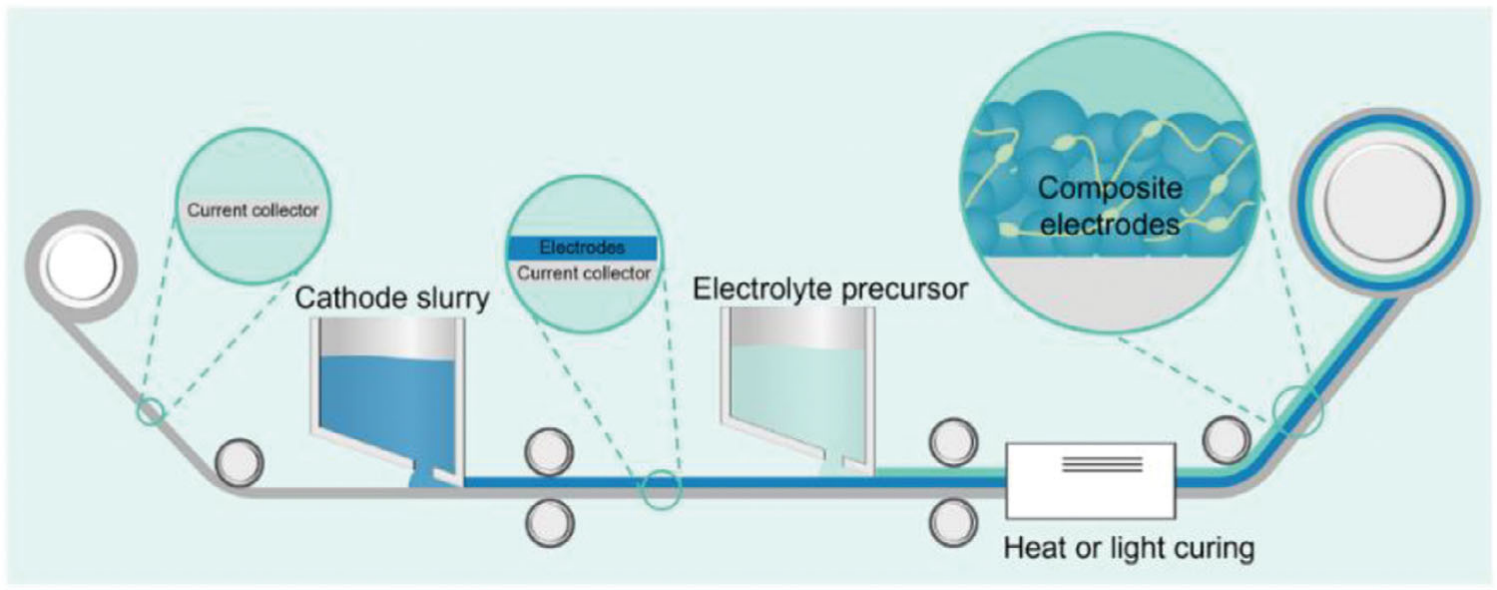
Composite electrode architecture and fabrication. Fabrication process: sequential slot‐die coating of cathode slurry and electrolyte precursor onto current collector, UV or thermal curing to form integrated electrode structure, and final conditioning for cell assembly. This roll‐to‐roll compatible process enables scalable manufacturing.

The benefits of this design are twofold. First, it significantly reduces interfacial resistance by maximizing contact area between the electrolyte and the active material.^[^ ^121^ ^]^ Since the ion transport must occur across these interfaces during charge and discharge, intimate contact is critical to ensuring fast kinetics and minimizing polarization. The flexible, soft nature of polymers allows them to fill voids and conform to particle surfaces, creating a continuous ion‐conducting pathway and mitigating issues such as particle detachment or interfacial void formation that plague brittle solid/solid interfaces (**Figure** **10**).

Second, composite electrodes simplify cell integration and manufacturing, aligning more closely with established slurry‐coating and calendaring techniques used in the LIB production.^[^ ^122^ ^]^ This compatibility reduces the need for high‐temperature sintering, stack pressing, or other mechanically aggressive processing steps typically associated with ceramic‐based systems. Furthermore, polymer‐based systems can accommodate roll‐to‐roll fabrication and multilayer lamination (Figure 10), offering a scalable path toward high‐throughput production.

As polymer electrolyte systems continue to evolve, their role in enabling efficient, scalable composite electrode designs is becoming increasingly central. These architectures not only improve performance but also streamline the path toward manufacturable and commercially viable solid‐state battery technologies.

### 4.3 Electrolyte Layer Processing

Processing the electrolyte layer is a critical step in the fabrication of polymer‐based SSBs, influencing not only the electrochemical performance but also the mechanical integrity and manufacturability of the cell. One of the key advantages of polymer‐based electrolytes lies in their solution processability, which allows for low‐temperature, scalable fabrication methods that are difficult or impossible with rigid ceramic electrolytes.^[^ ^123^ ^]^

**Figure 11:**
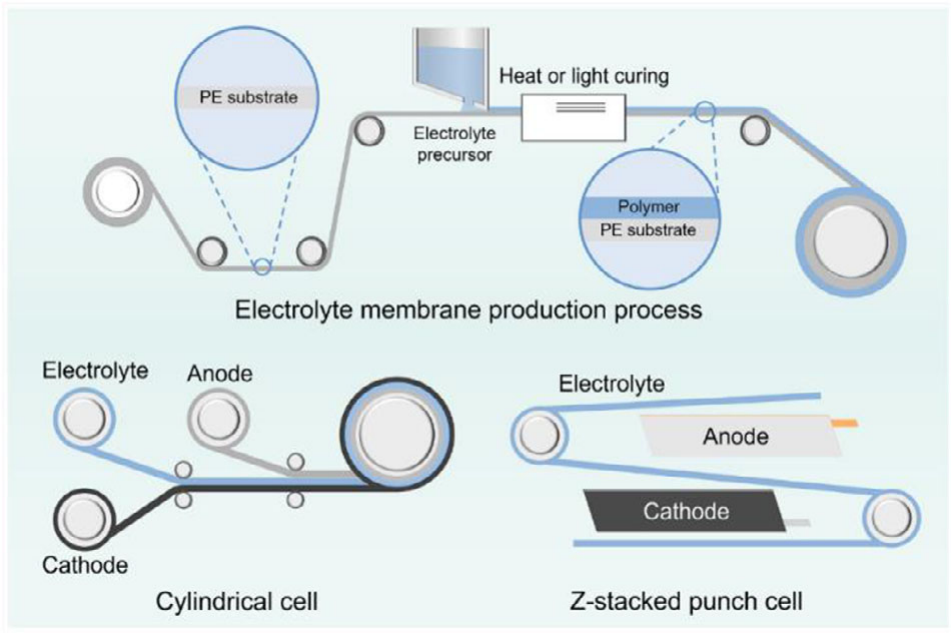
Scalable fabrication of polymer electrolyte membranes and cell integration. The electrolyte precursor solution is doctor‐blade cast onto the PE substrate and thermally cured to form a continuous membrane, followed by winding for cylindrical cells or lamination for pouch cell assembly, enabling compatibility with large‐scale battery production.

Polymer‐based electrolytes can be cast as membranes from solution or melt‐processed into freestanding membranes.^[^ ^124^ ^]^ This versatility supports a range of manufacturing routes, including doctor blading, slot‐die coating, extrusion, and even printing techniques. Unlike ceramic electrolytes, which often require high‐temperature sintering and stringent atmosphere control, polymer membranes can be processed under mild conditions, making them more compatible with the existing LIB production infrastructure (**Figure** **11**).

The thickness and uniformity of the electrolyte layer are key factors influencing cell resistance and energy density. Polymers allow for the fabrication of sub‐10 µm membranes that maintain mechanical flexibility and ionic conductivity, enabling high energy density while preserving solid/solid contact integrity.^[^ ^125^ ^]^ Techniques such as solvent evaporation, phase inversion, and hot pressing are commonly used to fine‐tune membrane morphology and porosity, thereby optimizing ionic transport and mechanical strength.

Furthermore, polymer–inorganic composite electrolytes present additional opportunities in processing. By dispersing ceramic particles into a polymer matrix, one can tailor rheological properties for improved membrane formation, reduce shrinkage during drying, and enhance dimensional stability.^[^ ^126^ ^]^ These systems also allow for layer‐by‐layer deposition or co‐extrusion with electrodes, facilitating monolithic cell architectures.

Efforts are also underway to develop in situ polymerization strategies, where a liquid monomer or oligomer precursor is coated or injected into the cell stack and subsequently polymerized thermally or photochemically to form the solid electrolyte layer.^[^ ^127^ ^]^ This method offers excellent conformality and integration, particularly at interfaces, and eliminates the need for post‐assembly lamination or compression.

Overall, the processing flexibility of polymers represents a major manufacturing advantage, offering a pathway toward low‐cost, scalable, and high‐throughput SSB production. However, processes such as UV treatment and solvent recovery systems (e.g., NMP) require continuous improvement to fully comply with OSHA and REACH regulations. Continued innovation in membrane casting, composite design, and in situ polymerization will be key to realizing the full potential of polymer electrolytes in commercial applications.

### 4.4 Electrode–Electrolyte Assembly

The assembly of electrodes and electrolyte layers is a critical stage in the fabrication of polymer‐based SSBs, influencing both electrochemical performance and mechanical reliability. Polymer electrolytes offer substantial advantages in this context due to their conformability, flexibility, and processing compatibility with conventional LIB manufacturing techniques.

Unlike ceramic electrolytes, which often require high‐pressure stack pressing or sintering to achieve sufficient interfacial contact, polymer electrolytes can be laminated or cast directly onto electrode surfaces. This low‐temperature, pressure‐free integration enables intimate and uniform contact at the electrode/electrolyte interface, a key factor in minimizing interfacial resistance and ensuring stable cycling performance.

Two primary approaches are used in electrode–electrolyte assembly for polymer‐based systems:

Ex situ lamination, where preformed polymer electrolyte membranes are pressed or thermally bonded onto the electrode surface.^[^ ^128^ ^]^ This method benefits from precise control over layer thickness and material quality but may require surface pretreatment (e.g., plasma activation or solvent swelling) to ensure good adhesion and ionic contact.

In situ polymerization, where the electrolyte is applied in liquid form—either as a dissolved polymer or as a monomer/oligomer precursor—onto the electrode, followed by curing or solvent evaporation to form the solid electrolyte layer.^[^ ^129^ ^]^ This approach allows the electrolyte to fill surface irregularities and maintain conformal contact, making it especially attractive for complex or porous electrode structures. Concurrently, low‐toxicity and high‐efficiency initiators were developed,^[^ ^130^ ^]^ alongside the implementation of a UV/thermal dual‐initiation system and a gradient polymerization strategy to accelerate polymerization kinetics and achieve near‐complete monomer conversion.^[^ ^131^ ^]^ These innovations collectively address the challenge of electrolyte solidification uniformity, thereby significantly improving battery consistency and production yield.

The ductility of polymers also helps accommodate volume changes during cycling, particularly with high‐capacity anodes like lithium metal or silicon.^[^ ^132^ ^]^ This mechanical compliance reduces the risk of interfacial delamination and maintains contact integrity over extended cycling, improving long‐term cell stability.

Advanced techniques such as hot pressing, solvent‐assisted bonding, and multilayer extrusion are also being explored to optimize the electrode/electrolyte interface. The choice of assembly method often depends on the specific polymer system, cell design, and application requirements, but across all approaches, the adaptability of polymer electrolytes provides a decisive advantage over brittle, rigid alternatives.

Ultimately, polymer‐based assembly strategies hold strong promise for enabling scalable, high‐performance SSB manufacturing, with significant reductions in interfacial resistance and processing cost.

### 4.5 Scalability and Industrial Integration

For any next‐generation battery technology to achieve widespread adoption, scalability, and compatibility with industrial manufacturing processes are essential. In this regard, polymer‐based SSBs are uniquely well‐positioned due to their processing flexibility, low‐temperature fabrication, and compatibility with roll‐to‐roll production techniques—a distinct contrast to the high‐cost, high‐temperature methods required for ceramic systems.

Polymer‐based electrolytes can be processed using methods already common in the LIB manufacturing, such as slurry casting, extrusion, film lamination, and slot‐die coating.^[^ ^133^ ^]^ These scalable, continuous processes enable high‐throughput production of electrolyte membranes, composite electrodes, and multilayer assemblies, reducing the need for costly custom equipment or energy‐intensive post‐processing steps like sintering or high‐pressure pressing.

Another major advantage is the ability to implement dry‐room or ambient‐condition fabrication, depending on the polymer chemistry. This stands in contrast to sulfide‐based electrolytes, which often require stringent moisture control and specialized environments. Polymers thus reduce facility overhead and simplify integration into existing gigafactory lines.

Moreover, the mechanical flexibility of polymers allows for the fabrication of thin, conformal, and stackable layers, opening the door to monolithic cell architectures and high‐energy‐density pouch formats. These form factors are already familiar to battery manufacturers and can be rapidly adapted to polymer‐based systems. In addition, the ability to integrate polymer electrolytes via in situ polymerization or co‐curing streamlines manufacturing by reducing the number of discrete processing steps and improving interfacial contact without compromising throughput.

Challenges remain, including ensuring uniform layer thickness at industrial speeds, managing solvent recovery in large‐scale operations, and maintaining interfacial stability during fast assembly. However, these are largely engineering rather than fundamental barriers, and active development is underway to address them through automation, real‐time quality control, and modular process design.

Polymer precursors are abundant and industrially mature, supported by well‐established chemical supply chains. This abundance, combined with the inherent versatility of polymer chemistry, provides a significant advantage in transitioning to polymer‐based SSBs. The ability to leverage these existing supply chains ensures a smoother, more cost‐effective shift, facilitating the rapid scaling of production to meet growing demand.

Moreover, the lower raw material costs, simplified processing techniques, and minimal factory retooling needed for polymer‐based systems make them a highly attractive choice in a cost‐sensitive market. Polymer‐based SSBs present a compelling economic advantage due to their compatibility with the existing manufacturing infrastructure. This reduces capital expenditure for factory modifications while enabling high‐volume, high‐throughput production. Taken together, these factors establish polymer‐based SSBs as the clear frontrunner in terms of cost competitiveness, positioning them for widespread adoption as the industry seeks both performance enhancements and cost reductions.

Once confined to the laboratory, polymer‐based solid‐state batteries are now advancing rapidly toward industrial adoption. For example, 50‐m‐long and 0.3‐m‐wide rolls of PEO‐Mg‐Al‐LiTFSI electrolyte membranes were fabricated via an industrial‐scale continuous slurry‐casting process, in which the slurry was coated onto a polyethylene (PE) substrate.^[^ ^68^ ^]^ Using these membranes, 20 Ah all‐solid‐state polymer batteries were successfully assembled, exhibiting high capacity, excellent coulombic efficiency, and stable cycling performance—demonstrating strong practical potential. In another approach, in situ polymerization within porous polypropylene (PP) separators enabled effective pore filling, yielding membranes that remained dry, nonsticky, and processable into continuous rolls suitable for assembling large single cells exceeding 300 Ah.^[^ ^121^ ^]^ By leveraging existing electrode and manufacturing technologies,^[^ ^46^ ^]^ the large‐scale production of polymer‐based solid‐state batteries is becoming increasingly feasible.

**Figure 12:**
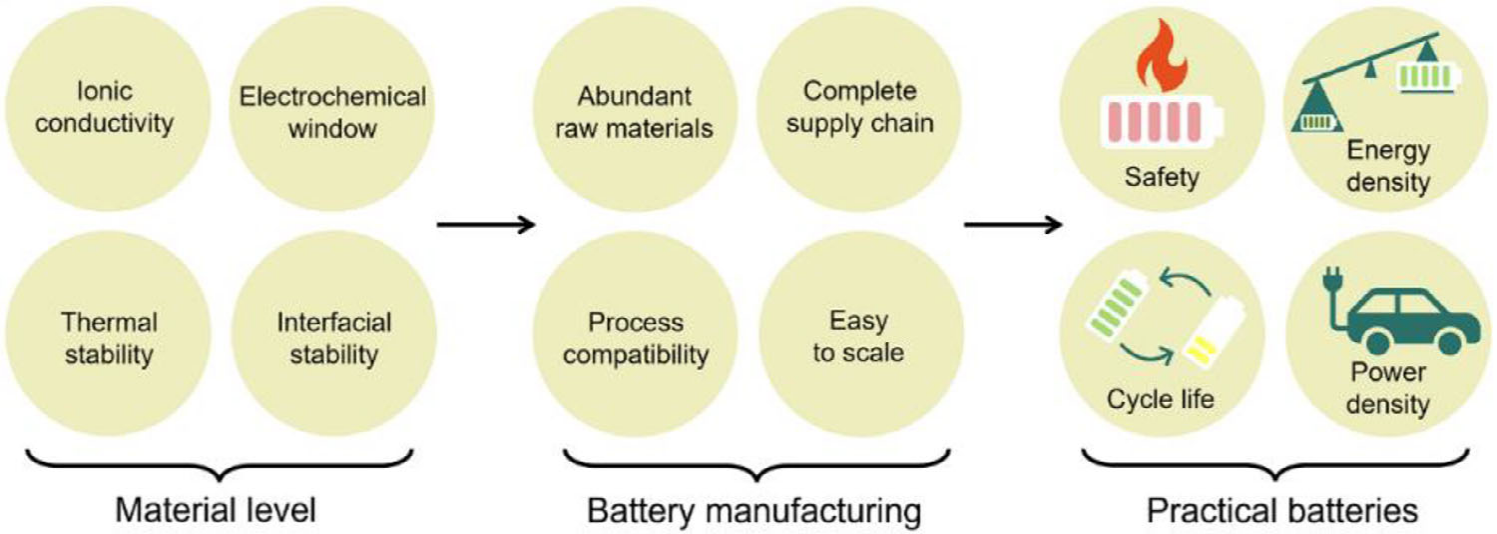
Scalability and industrial integration of polymer‐based SSBs: from lab‐level research to large‐scale production.

In summary, the industrial integration of polymer‐based SSBs is not only feasible but also strategically advantageous (**Figure** **12**). By utilizing existing manufacturing infrastructure and minimizing the need for disruptive process changes, polymer electrolytes provide a clear and scalable pathway from laboratory innovations to large‐scale commercial production.

## 5 Conclusions

Polymers offer an optimal balance between manufacturability, safety, and performance, making them a standout choice for solid‐state batteries. Their soft, flexible nature ensures superior interfacial contact with lithium metal anodes and cathodes, which is crucial for mitigating interfacial side reactions and enhancing long‐term cycling stability—key challenges for the SSB technology. The versatility of polymer chemistry allows for precise tailoring of properties such as ionic conductivity, mechanical strength, and electrochemical stability, enabling further improvements as research progresses.

In addition, polymers significantly reduce capital expenditure for battery manufacturers. Unlike oxide and sulfide electrolytes, which require high‐temperature sintering or dry‐room environments, polymer electrolytes can be processed under milder conditions, simplifying production and lowering operational costs. As research continues to improve their conductivity and long‐term durability, polymers are increasingly positioned to become the choice for commercial SSB deployment.

In conclusion, while oxide and sulfide electrolytes have their specific advantages, their practical limitations in terms of processing complexity, stability, and cost make them less suitable for mass adoption. Polymer electrolytes, with their inherent versatility, processability, and scalability, present the most realistic path toward the widespread commercialization of safe, high‐performance, and economically viable solid‐state batteries.

Polymers are not just part of the race—they are leading it.

## Conflict of Interest

The authors declare no conflict of interest.
